# Upregulation of IL-17A/F from human lung tissue explants with cigarette smoke exposure: implications for COPD

**DOI:** 10.1186/s12931-014-0145-7

**Published:** 2014-11-27

**Authors:** Ying Chang, Laila Al-Alwan, Sama Alshakfa, Severine Audusseau, Andrea Karen Mogas, Fazila Chouiali, Parameswaran Nair, Carolyn J Baglole, Qutayba Hamid, David H Eidelman

**Affiliations:** Meakins-Christie Laboratories and Respiratory Division, McGill University Health Centre and Department of Medicine, McGill University, Montreal, Quebec Canada; Center for Translational Medicine, The Key Laboratory of Biomedical Information Engineering of Ministry of Education, School of Life Science and Technology and Frontier Institute of Science and Technology, Xi’an Jiaotong University, Xi’an, China; Firestone Institute for Respiratory Health, St. Joseph’s Healthcare and Department of Medicine, McMaster University, Hamilton, Ontario Canada

**Keywords:** COPD, IL-17, Cigarette smoke, Tissue explants

## Abstract

**Background:**

Chronic obstructive pulmonary disease (COPD) is an inflammatory disorder marked by relative resistance to steroids. The IL-17 superfamily, which mediates cross-talk between the adaptive and innate immune systems, has been associated with diminished responses to steroids. Increasing evidence supports elevated IL-17 expression in the lung of COPD subjects. However, whether cells of the immune system (systemic) and/or local lung cells are contributing to the elevated IL-17 remains unclear. To address this issue, we utilized a human parenchymal lung tissue explant culture system with cigarette smoke exposure to investigate the expression of IL-17 and the mechanisms involved.

**Methods:**

Parenchymal lung tissue removed from 10 non-COPD and 8 COPD patients was sectioned and cultured with different concentrations of cigarette smoke extract (CSE) for 3 or 6 hours. Tissue viability was evaluated by LDH (lactate dehydrogenase) in culture supernatants. Western blot and real-time PCR were performed to evaluate IL-17A/F expression. To investigate the mechanisms, pharmacological inhibitors for MAPK p38, ERK1/2, NF-κB and PI3K pathways were added into the culture media.

**Results:**

No tissue damage was observed after the cigarette smoke exposure for 3 h or 6 h compared with the control media. At the protein level, the expression of both IL-17A (2.4 ± 0.6 fold) and IL-17 F (3.7 ± 0.7 fold) in the tissue from non-COPD subjects was significantly increased by 5% of CSE at 3 h. For COPD subjects, IL-17A/F expression were significantly increased only at 6 h with 10% of CSE (IL-17A: 4.2 ± 0.8 fold; IL-17 F: 3.3 ± 0.8 fold). The increased expression of IL-17A/F is also regulated at the mRNA level. The inhibitors for NF-κB and PI3K pathways significantly inhibited CSE-induced IL-17A/F expression from lung tissue of non-COPD subjects.

**Conclusions:**

We found the evidence that the expression of both IL-17A and IL-17 F is increased by the cigarette smoke exposure in explants from both non-COPD and COPD subjects, supporting that local lung cells contribute IL-17 production. The elevated IL-17A/F expression is dependent on NF-κB and PI3K pathways. These observations add to the growing evidence which suggests that Th17 cytokines play a significant role in COPD.

## Background

Chronic obstructive pulmonary disease (COPD) is a progressive, irreversible chronic inflammatory disorder that is caused predominantly by cigarette smoking and is one of the leading causes of mortality globally [1]. The inflammatory response in the lungs of COPD patients is strongly linked to tissue destruction and alveolar airspace enlargement, which lead to disease progression [2]. Recent findings concerning the innate and acquired immune responses in COPD have led to the suggestion that there is an autoimmune component to its pathogenesis [3,4]. The mechanism by which cigarette smoke causes COPD in susceptible individuals is complex, but involves both aberrant, chronic inflammation coupled with the loss of lung structural cells due to heightened apoptosis.

The IL-17 cytokine superfamily triggers production of numerous chemokines, resulting in neutrophil and macrophage recruitment and subsequent pathogen clearance. IL-17 is key to defense against bacteria and fungi, mediating cross-talk between the adaptive and innate immune systems [5]. IL-17A can influence expression of mucin (MUC5AC), a hallmark of chronic airway diseases including COPD, in human bronchial epithelial cells [6]. Furthermore, transgenic over expression of IL-17A in the alveoli of murine lung induces inflammation with a COPD-like phenotype [7]. Elevated IL-17A secretion has been reported to be present in the bronchial mucosa of COPD patients [8]. In addition, given the possible importance of IL-17A in autoimmunity [7], it is of interest to note the growing evidence that autoimmunity may contribute to the pathogenesis of COPD [3,9]. Our previous study has demonstrated the elevated IL-17A/F expression in airways of COPD patients compared to control subjects, and CD8^+^ T cells are a major cellular source [10]. However, whether the elevation of IL-17 is dependent on the local lung immune and structural cells or the recruitment of systemic immune cells remains unclear.

To address these questions we utilized tissue explants, widely used in cellular biology because of the preservation of histotypic relationship between cells in the tissue without any disturbance of the cellular or tissue architecture caused by other methods utilizing enzymatic, chemical or mechanical separation [11]. By exposing explanted lung tissue to cigarette smoke, we can detect whether cigarette smoke itself has an effect on local lung immune and structural cells. Using this approach, we found that cigarette smoke exposure promoted IL-17A/F expression of lung explants from both COPD and non-COPD subjects quickly, suggesting parenchymal tissue is an important source of IL-17A/F in individuals who smoke.

## Methods

### Subjects

Parenchymal lung tissue was removed from 10 non-COPD and 8 COPD patients undergoing thoracic surgery at St. Joseph’s Healthcare Hamilton, Ontario, with the collaboration of the Division of Thoracic Surgery and the Department of Pathology. The COPD patients were eligible for this study if they met the following criteria: age ≥50 and ≤76 years; smoking history (≥20 pack-years); post-bronchodilator FEV_1_ ≥ 25% of predicted value and post-bronchodilator FEV_1_/forced vital capacity (FVC) ≤0.70; no history of asthma, atopy (as assessed by an allergy skin prick test during screening) or any other active lung disease. Patients on home oxygen or with raised carbon dioxide tension (>44 mmHg), α_1_-antitrypsin deficiency, recent exacerbation (in the last 4 weeks), an uncontrolled medical condition or hypersensitivity to inhaled corticosteroids and bronchodilators were not eligible for the study. Patient characteristics are in Table 1. This study was conducted in accordance with the amended Declaration of Helsinki. Local institutional review boards or independent ethics committees approved the protocol, and written informed consent was obtained from all patients. The experimental procedures were performed with ethical approval from the Research Ethics Boards of St. Joseph’s Healthcare, McMaster University.

**Table 1:** **Clinical characteristics of COPD and control subjects**

	**COPD**	**Controls**
Number	8	10
Age	63 ± 9	69 ± 8
Male/Female	3/5	6/4
Current/ex-smokers	5/3	2/7
Post-BD FEV1% predicted	78 ± 13	95 ± 17
GOLD Stage		
I	4	-
II	3	-
III-IV	1	-
Respiratory Medication		
SABD	3	-
LABD	3	-
ICS	2	-
Combination (LABD + ICS)	2	-
Theophylline	0	-

Data are presented as mean ± SD. BD, bronchodilator; FEV1, forced expiratory volume in 1 s; TLCO, Transfer Factor of the Lung for Carbon Monoxide; SABD, short-acting bronchodilators; LABD, long-acting bronchodilators; ICS, inhaled corticosteroids.

### Tissue explants

**Figure 1:**
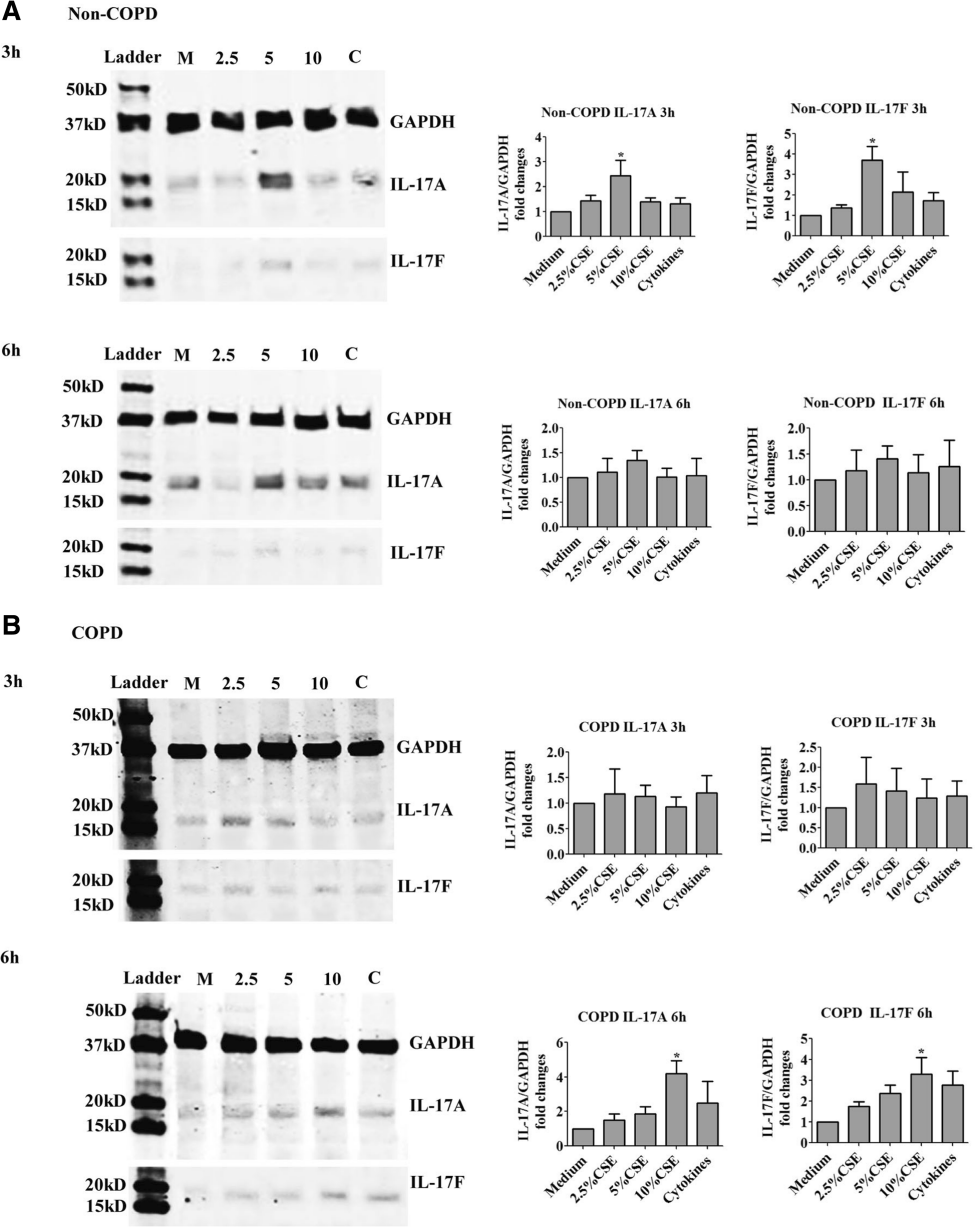
**Protein expression of IL-17A/F in human lung explants exposed to cigarette smoke extract.** Human lung explants from Non-COPD **(A)** and COPD subjects **(B)** were cultured with different concentration of cigarette smoke extract for 3 h or 6 h. The expression of IL-17A/F was detected by western blot. The results are presented as means ± SEM. The combination of cytokines (labeled as C) was used as a control. N = 10 for Non-COPD subjects and N = 8 for COPD subjects. CSE: Cigarette smoke extract. Cytokines: 20 ng/ml each of IL-1β, IL-6 and IL-23. ^*^P < 0.05 compared with medium control. The label “M”, “2.5”, “5” and “10” represent medium, 2.5%, 5% and 10% of CSE respectively.

Tissue specimens were immediately placed in Perfadex lung transplant solution on ice for transportation. Upon arrival, the tissue was placed in Bicarbonate Buffered Culture Medium (BCM) containing with 20 ml/L of amino acid supplement, 10 ml/L of sodium pyruvate, 10 ml/L of vitamin supplement, 50 μg/ml of gentamycin, 0.1 μg/ml of insulin, 0.1 μg/ml of vitamin A and 0.1 μg/ml of Hydrocortisone. Under sterile conditions, the tissue was sectioned into approximately 1 mm thick slices and placed on 0.4-μm inserts (Millipore, Bedford, Mass) in different concentrations of CSE (2.5%, 5% and 10%), a cytokine combination (labeled as cytokines in Figure 1) consisting of 20 ng/ml of IL-1β, IL-6 and IL-23 each, or media alone. The explants were incubated in 5% CO_2_/95% air at 37°C for 3 or 6 hours after which they were processed for protein and RNA.

In separate experiments, we used pharmacological inhibitors against cellular pathways known to contribute to IL-17 expression. Specifically, we used the p38 MAPK inhibitor BIRB796 (0.1 μM; Axon Medchem BV, Groningen, The Netherlands) [12], the extracellular signal-regulated kinase (ERK)1/2 mitogen-activated protein kinase (MAPK) inhibitor PD184352 (2 μM; US Biological, Swampscott, MA, USA) [13], the nuclear factor κB (NF-κB) inhibitor helenalin (1 μM; Enzo Life Sciences, Ann Arbor, MI, USA) [14], and the phosphoinositide 3-kinase (PI3K) inhibitor PI103 (5 μM; Cayman Chemical, Ann Arbor, MI, USA) [15]. In each case, explants were incubated with the inhibitor for 1 h at 37°C prior to the CSE or cytokine cocktail. All results were compared to the corresponding vehicle control (DMSO).

### Preparation of cigarette smoke extract

3R4F reference cigarettes with a filter were obtained from the Kentucky Tobacco Research Council (Lexington, KT) and CSE was generated as previously described [16]. An optical density of 0.65 (320 nm) was considered to represent 100% CSE [16]. This CSE preparation was diluted to the appropriate concentration in BCM.

### LDH assay

Lung tissue explants were cultured with increasing concentrations of CSE (2.5%, 5% and 10%) for 3 h, 6 h, 12 h and 24 h. The culture supernatants were collected for lactate dehydrogenase (LDH) detection using a Cytotoxicity LDH Detection Kit (Clontech, Mountain View, CA, USA). The results are presented as OD value and have been corrected by total protein concentration assayed by BCA protein assay kit (Thermo scientific, Rockford, IL, USA).

### Western blot

At the end of exposure, the explants were stored in −80°C with RNA later (Ambion, Grand Island, NY, USA). Tissues were homogenized, lysed, and the lysate (10 μg) was loaded on 10% acrylamide SDS-PAGE NEXT GEL (Amresco, Solon, Ohio), followed by transfer to nitrocellulose membranes (Bio-Rad, Hercules, Calif). The blots were then blocked for 1 h at room temperature and incubated overnight at 4°C with antibodies specific for IL-17A (R&D Systems, Minneapolis, MN, USA), IL-17 F (Santa Cruz Biotechnology, Santa Cruz, Calif) and GAPDH (Millipore, Temecula, USA). After washing (0.1% Tween-20/PBS), the membranes were incubated with a 1:15,000 dilution of IRDye 800 donkey anti-goat IgG and IRDye 680 goat anti-mouse IgG (Rockland) in blocking buffer and analyzed with an Odyssey IR scanner using Odyssey imaging software 3.0 (LI-COR Biosciences, Inc).

### Immunohistochemistry staining

The immunohistochemistry staining for IL-17A and IL-17 F (R&D systems) was performed on paraffin-embedded human lung tissue sections as previously described [17]. Sections were also stained with isotype control Ab and a primary anti-IL-17A or anti-IL-17 F Ab was preadsorbed with a 5-fold mass excess of the immunizing peptide (human IL-17A or IL-17 F recombinant protein; R&D systems) for 1 h at 4°C.

### Quantitative Reverse Transcription-PCR

Quantitative RT-PCR for IL-17A, IL-17 F and glyceraldehyde-3-phosphate dehydrogenase (GAPDH) was performed as previously described [10]. The IL-17A/F mRNA expression was normalized to GAPDH and compared using the δδCt method. All results were expressed as relative quantity (RQ) compared to medium controls at 3 h or 6 h.

### Statistical analysis

All data are presented as means ± SEM. For the results of western blot detecting IL-17A/F expression, statistical analysis was performed using ANOVA followed by the post-hoc Bonferroni test. For the results of Real-time PCR and signaling pathway experiments, statistical analysis was performed using t test. P values of less than 0.05 were regarded as statistically significant. Statistical analysis was performed with GraphPad Instat 3 software (GraphPad Software, Inc, La Jolla, Calif).

## Results

### Viability of lung tissue explants

**Figure 2:**
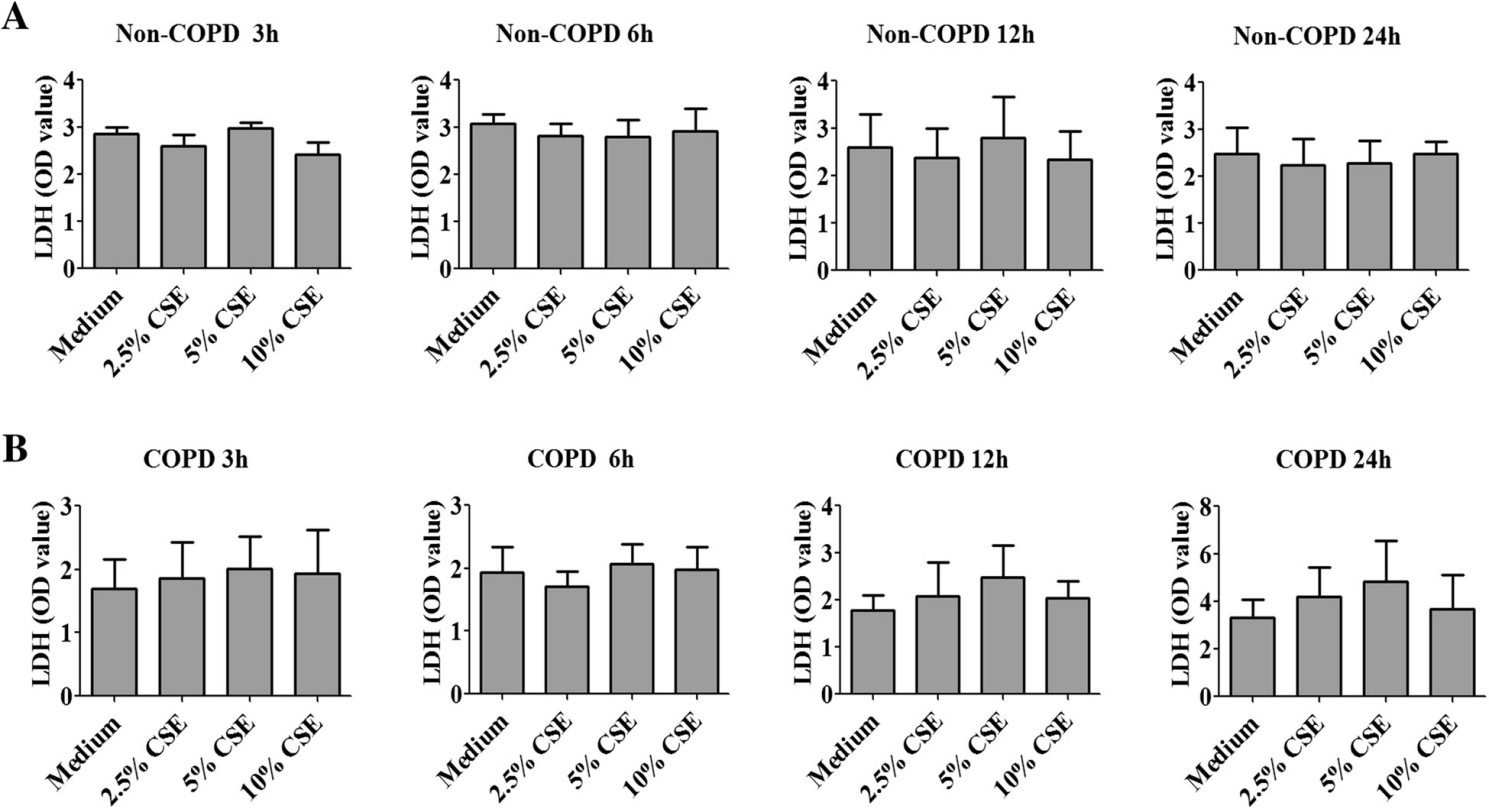
**Viability of human lung explants exposed to cigarette smoke extract (CSE).** Human lung explants were cultured with different concentration of cigarette smoke extract for 3 h, 6 h, 12 h and 24 h. The level of lactate dehydrogenase (LDH) in culture supernatant was assayed. The results are presented as OD value, were corrected for total protein concentration.

LDH release in culture supernatant is a reliable index of cell injury [18-20], which we used to evaluate tissue viability after cigarette smoke exposure. In non-COPD subjects, no apparent damage was observed in cigarette smoke-exposed tissue compared to that of medium control at any time points examined (Figure 2A). On the contrary, damage occurred in the cigarette smoke exposure conditions from 12 h time point in tissues from COPD subjects (Medium OD value: 1.8 ± 0.3; 5% CSE OD value: 2.5 ± 0.7) (Figure 2B). Thus, we chose shorter time points- including 3 h and 6 h- for the remaining studies.

### IL-17A/F expression in lung tissue

The protein expression of IL-17A/F in lung tissue explants was first evaluated by western blot. The expression of both IL-17A (2.4 ± 0.6 fold increase, P < 0.05) and F (3.7 ± 0.7 fold increase, P < 0.05) in the tissue from non-COPD subjects was significantly increased after exposure to 5% of CSE at 3 h, which returned to baseline by 6 h (Figure 1A). In lung tissue from COPD subjects, a significant increase in IL-17A/F expression was observed at 6 h with 10% of CSE (IL-17A: 4.2 ± 0.8 fold increase; IL-17 F: 3.3 ± 0.8 fold increase, P < 0.05, Figure 1B). Cytokines known to induce Th17 differentiation including IL-1β, IL-6 and IL-23 were used as controls [21,22]. These cytokines did not significantly increase the expression of IL-17A/F of lung explants from either non-COPD or COPD subjects (Figure 1), which may due to the impaired effect of these cytokines on the structural cells.

**Figure 3:**
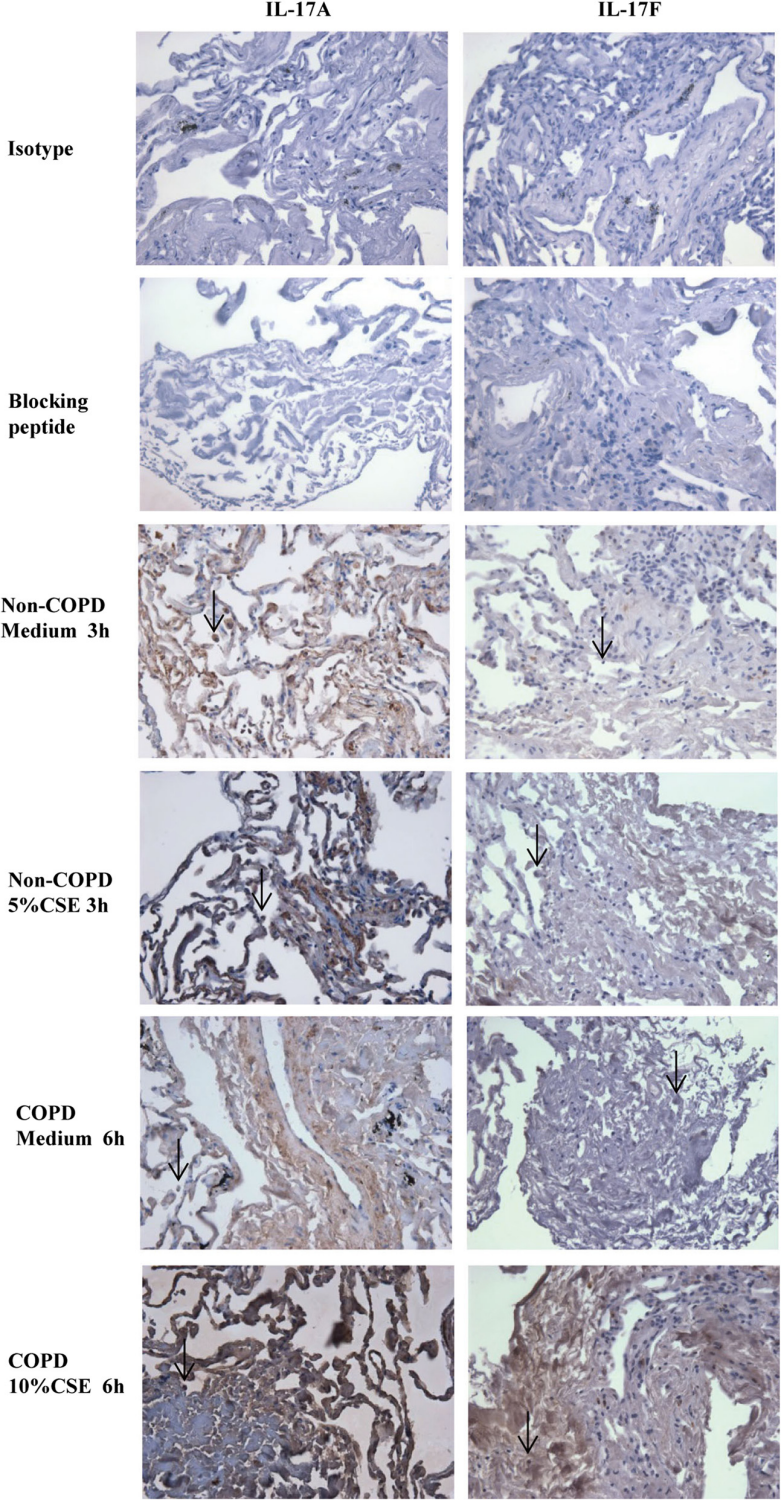
**Immunohistochemistry staining for IL-17A/F in human lung explants exposed to cigarette smoking medium.** Human lung explants from Non-COPD and COPD subjects were cultured with 5% or 10% of CSE for 3 h or 6 h respectively. Brown color staining represents the positive signal for IL-17A/F. The arrows indicate the local immune cells in the lung tissue. Representative images from 5 Non-COPD and 4 COPD subjects are shown. Magnification: 400X.

IL-17A and IL-17 F can be produced by both immune cells and structural cells [23,24]. Our finding that production of these cytokines is increased in cultured lung slices exposed to CSE, raises the question as to which cells are responsible for this increased expression. Using immunohistochemistry staining to identify the source of IL-17A/F in explants, we observed that both local structural and immune cells expressed IL-17A/F (Figure 3). The arrow in the picture Non-COPD medium indicates the expression of IL-17 F in the local immune cells of lung tissue. The absence of color may indicate the low expression of IL-17 F in these cells without CSE stimulation, while it appeared with CSE stimulation. This finding suggests that CSE induces IL-17A/F expression from lung parenchyma under the absence of systemic immune cell recruitment.

**Figure 4:**
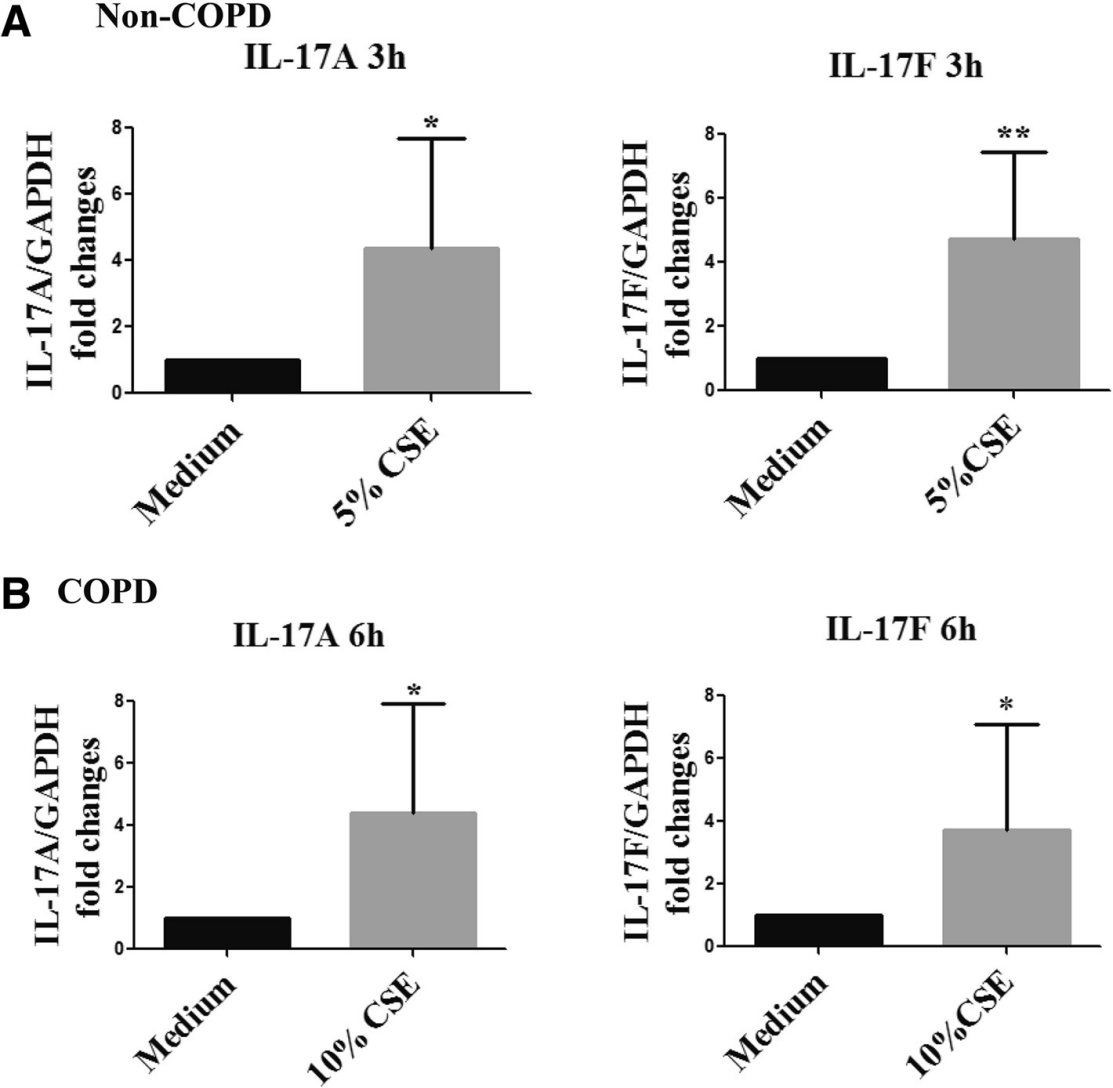
**mRNA expression of IL-17A/F in human lung explants exposed to cigarette smoking medium. (A)** Human lung explants from Non-COPD subjects were cultured with 5% of CSE for 3 h. N = 10 subjects. **(B)** Human lung explants from COPD subjects were cultured with 10% of CSE for 6 h. N = 8 subjects. The mRNA expression of IL-17A/F was detected by real-time PCR. CSE: Cigarette smoke. ^*^P < 0.05, ^**^P < 0.01 compared with medium control.

The mRNA expression of IL-17A/F in lung tissue explants under optimal CSE concentration and exposure time (Non-COPD: 5% of CSE for 3 h; COPD: 10% of CSE for 6 h) was examined by real-time PCR. The IL-17A/F mRNA expression was also significantly elevated as those observed on protein level (Figure 4). These results suggest that the elevated IL-17A/F expression under CSE exposure occurred at the transcriptional level.

### Signaling pathways involved in CSE-induced IL-17 A/F expression

**Figure 5:**
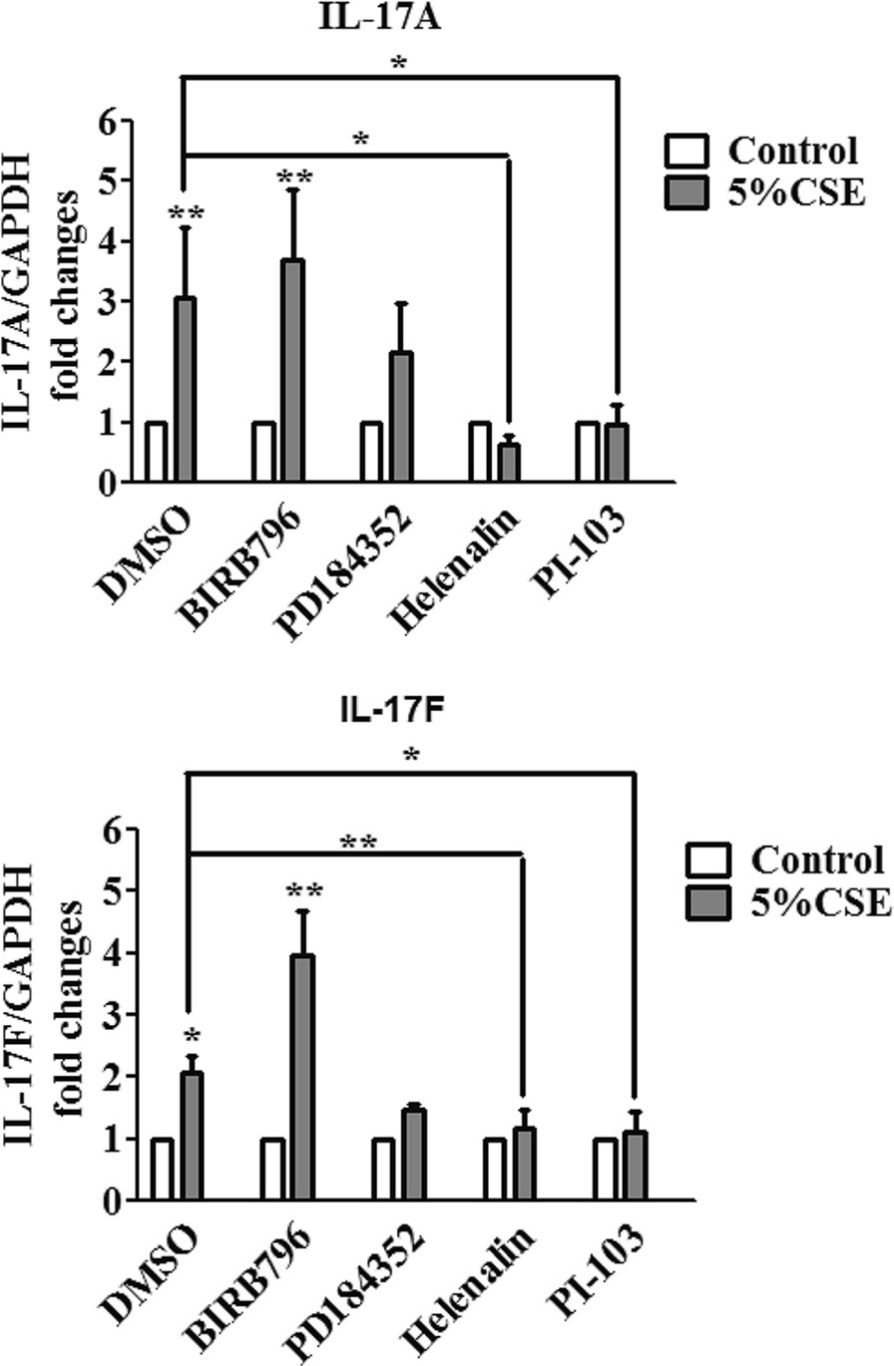
**Signaling pathways involved in CSE-induced IL-17 A/F expression from human lung explants.** The explants were incubated with the p38 MAPK inhibitor BIRB796 (0.1 μM), the ERK1/2 inhibitor PD184352 (2 μM), the NF-κB inhibitor helenalin (1 μM), and the PI3K inhibitor PI103 (5 μM) for 1 h at 37°C prior to the exposures. The explants from Non-COPD subjects were cultured with 5% of cigarette smoke extract (CSE) for 3 h. All results were compared to the corresponding vehicle control DMSO. The results are presented as means ± SEM. N = 7 subjects. ^*^P < 0.05, ^**^P < 0.01.

We next examined the signaling pathway involved in the elevated IL-17A/F expression of lung tissue explants from Non-COPD subjects with CSE exposure. The specific pharmacological inhibitor for MAPK p38, ERK1/2, NF-κB and PI3K pathways was used. These inhibitors did not cause the toxicity of tissue, which was examined by LDH level in the culture supernatant (data not shown). The protein expression of IL-17A/F was evaluated by western blot. The inhibitors for NF-κB (Helenalin) and PI3K (PI-103) significantly attenuated CSE-induced IL-17A (DMSO: 3.1 ± 1.2 folds; Helenalin: 0.7 ± 0.2 fold, P < 0.05; PI-103: 1.0 ± 0.3 fold, P < 0.05) and IL-17 F (DMSO: 2.0 ± 0.2 folds; Helenalin: 1.2 ± 0.2 fold, P < 0.01; PI-103: 1.1 ± 0.3 fold, P < 0.05) expression from lung tissue of non-COPD subjects (Figure 5). However inhibition of p38 (BIRB796) and ERK1/2 (PD184352) failed to reduce IL-17A/F expression induction by CSE. Interestingly, the MAPK p38 inhibitor BIRB796 significantly enhanced CSE-induced IL-17 F expression (3.9 ± 0.7 folds, P < 0.05 compared with DMSO, Figure 5). These results suggest that both NF-κB and PI3K pathway are involved in CSE-induced IL-17A/F expression from the lung tissue explants.

## Discussion

In the present study, we investigated the expression of IL-17A/F in explants of human lung tissue obtained from subjects with and without COPD. We found evidence that the expression of both IL-17A and IL-17 F is increased by the cigarette smoke exposure in explants from both non-COPD and COPD subjects. The local cells of lung tissue contributed to the heightened expression of these cytokines under the cigarette smoke exposure conditions. These observations add to the growing evidence which suggests that Th17 cytokines play a significant role in this disease.

We found parenchymal tissue from non-COPD subjects more rapidly induced IL-17A/F (at 3 h) in response to cigarette smoke, and that lower concentrations of CSE (5%) contributed to this induction. Contrary to this, a higher concentration of CSE (10%) and longer exposure times (6 h) were necessary to significantly elevate IL-17A/F expression in explants from COPD patients (Figure 1). This suggests that in COPD patients, the lung tissue and cells may have weaker ability to secrete significant levels of cytokines, which has been shown in our previous study concerning the secretary phenotype of CD8^+^ T cells in COPD patients [25]. All these may reflect the epigenetic changes in COPD that have led to phenotypic changes altering the responsiveness of cells. Another possibility contributing to the delayed expression of IL-17A/F in lung tissue from COPD subjects responded to CSE might lie in the interference from intrinsically increased oxidative stress in lung tissue under COPD status. In lung tissue of COPD subjects, the oxidant stress is increased [26], while a recent study showed that transient exposure to oxidative stress temporally could delay the activation of NF-κB [27], one of the signaling pathway involved in the IL-17A/F expression induced by CSE in the present study. Thus we suspect that the delayed expression of IL-17A/F in lung tissue of COPD subjects might due to the delayed NF-κB activation induced by increased oxidative stress.

A smoking-induced inflammatory reaction in the airways and lung parenchyma comprises neutrophils, alveolar macrophages and T cells, predominantly CD8^+^ T cells [28]. All of the aforementioned cells are the potential resource of IL-17 [5]. Parenchyma from COPD patients have more recruited cells (including T cells, monocytes and dendritic cells) and less structural cells (alveolar epithelial cells) than non-COPD tissue. However in this study we exposed the lung tissue explants to CSE and used the medium condition as the controls. Thus under the environment without intact circulation (no more cells will be recruited), the effect of CSE exposure on IL-17A/F expression in lung tissue was able to be observed. Nevertheless, the recruited immune cells in lung tissue of COPD patients have been restrained in explants and may contribute to the elevated production of IL-17A/F. In the present study, we demonstrated for the first time that CSE exposure could induce IL-17A/F expression from lung parenchyma in the absence of systemic immune cell recruitment, suggesting that CSE promotes expression of these cytokines in local lung cells. The notion that both local lung immune and structural cells could be important sources of IL-17 is supported by our recent publication demonstrating that CSE exposure enhanced IL-17A expression from mouse lung epithelial cells (MLE-12 cells), a distal bronchiolar and alveolar epithelial cell line [17]. Furthermore, more studies have shown that IL-17 is expressed by structural cells especially by epithelial cells. For example, IL-17 is expressed by tubular epithelial cells in renal transplant recipients [23], and IL-17 production has been described by Paneth cells, highly specialized epithelial cells of the small intestine [29].

We also explored the signaling pathways involved in CSE-induced IL-17A/F expression from lung explants. Cellular signaling pathways previously implicated in IL-17 production include the p38 MAPK [30], ERK1/2 MAPK [31], NF-κB and PI3K [32]. We found the IL-17A/F expression is dependent on both the NF-κB and PI3K pathways (Figure 5). The involvement of NF-κB and PI3K pathways in IL-17 induction has been shown in other inflammatory diseases like asthma [33] and rheumatoid arthritis [32], where IL-17 may play important pathogenic roles. It is noteworthy that the administration of p38 MAPK inhibitor BIRB796 enhanced CSE-induced IL-17A/F expression (Figure 5). This might be explained by the enhanced activity of NF-κB or PI3K pathway when p38 MAPK is inhibited. It has been reported that the p38 MAPK inhibitor SB203580 enhances NF-κB transcriptional activity via the ERK pathway [34].

## Conclusions

In conclusion, we demonstrated the direct effect of cigarette smoke on IL-17A/F expression from human lung parenchymal tissue by using an *ex-vivo* explant culture system. It indicates that elevated IL-17A/F in COPD is the product from not only the increased number of recruited inflammatory cells but also by the heightened expression of these cytokines in the local cells under the cigarette smoke exposure conditions. Thus, future exploration focusing on IL-17-targeted therapies in COPD is warranted.

## Abbreviations

COPD: Chronic obstructive pulmonary disease
CSE: Cigarette smoke extract
MAPK: Mitogen-activated protein kinase
ERK: Extracellular signal-regulated kinase
PI3K: Phosphoinositide 3-kinase
